# *AtHD2D* Gene Plays a Role in Plant Growth, Development, and Response to Abiotic Stresses in *Arabidopsis thaliana*

**DOI:** 10.3389/fpls.2016.00310

**Published:** 2016-03-31

**Authors:** Zhaofen Han, Huimin Yu, Zhong Zhao, David Hunter, Xinjuan Luo, Jun Duan, Lining Tian

**Affiliations:** ^1^College of Life Science, Northwest A & F UniversityYangling, China; ^2^Department of E-A Information Engineering, Liaoning Institute of Science and TechnologyBenxi, China; ^3^College of Forestry, Northwest A & F UniversityYangling, China; ^4^Southern Crop Protection and Food Research Centre, Agriculture and Agri-food CanadaLondon, ON, Canada; ^5^Key Laboratory of South China Agricultural Plant Genetics and Breeding, South China Botanical Garden, Chinese Academy of SciencesGuangzhou, China

**Keywords:** *AtHD2D*, expression and function, protein sub-cellular localization, plant development, stress tolerance

## Abstract

The histone deacetylases play important roles in the regulation of gene expression and the subsequent control of a number of important biological processes, including those involved in the response to environmental stress. A specific group of histone deacetylase genes, *HD2*, is present in plants. In *Arabidopsis, HD2s* include *HD2A, HD*2*B, HD*2*C*, and *HD*2*D*. Previous research showed that *HD2A, HD2B*, and *HD2C* are more related in terms of expression and function, but not *HD2D.* In this report, we studied different aspects of *AtHD2D* in *Arabidopsis* with respect to plant response to drought and other abiotic stresses. Bioinformatics analysis indicates that *HD2D* is distantly related to other *HD2* genes. Transient expression in *Nicotiana benthamiana* and stable expression in *Arabidopsis* of *AtHD2D* fused with *gfp* showed that *AtHD2D* was expressed in the nucleus. Overexpression of *AtHD2D* resulted in developmental changes including fewer main roots, more lateral roots, and a higher root:shoot ratio. Seed germination and plant flowering time were delayed in transgenic plants expressing *AtHD2D*, but these plants exhibited higher degrees of tolerance to abiotic stresses, including drought, salt, and cold stresses. Physiological studies indicated that the malondialdehyde (MDA) content was high in wild-type plants but in plants overexpressing *HD2D* the MDA level increased slowly in response to stress conditions of drought, cold, and salt stress. Furthermore, electrolyte leakage in leaf cells of wild type plants increased but remained stable in transgenic plants. Our results indicate that *AtHD2D* is unique among *HD2* genes and it plays a role in plant growth and development regulation and these changes can modulate plant stress responses.

## Introduction

In higher plants, transcriptional regulation of gene expression plays an important role in plant life. Post-translational modifications of histones, including acetylation, methylation, phosphorylation, ubiquitinylation, and adenosine diphosphateribosylation, result in modulating dynamic changes in chromatin configuration and gene activity (Berger, 2002; Reyes et al., 2002; Lee et al., 2005). Among these modifications, histone acetylation regulates a number of fundamental biological processes in plants. Histone acetylation levels are regulated by the action of histone acetyltransferases (HAT) that catalyze hyper acetylation and histone deacetylases (HDACs) that catalyze deacetylation by removing the acetyl group from the core histones. HDACs are an ancient group of enzymes that are widely spread in various organisms.

HDACs in plants can be divided into three families based on phylogenetic analysis. They are reduced potassium dependency 3(RPD3) or histone deacetylase 1 (HDA1), silent information regulator 2 (SIR2), and histone deacetylase 2 (HD2, also HD-tuins; Taunton et al., 1996; Wu et al., 2000; Tian and Chen, 2001; Pandey et al., 2002; Liu et al., 2014; Zhao et al., 2014). The HD2 family is specific to plants and is unrelated to the RPD3/HDA1 and SIR2 HDAC types (Sridha and Wu, 2006; Grandperret et al., 2014; Zhao et al., 2014). The first HD2 was isolated as an acidic nuclear phosphoprotein from maize embryos (Lusser et al., 1997). Expressed sequence tag (EST) homology searches identified four *HD2* genes in *Arabidopsis*, namely, *HD2A, HD2B, HD2C*, and *HD2D* (also known as *HDT1, HDT2, HDT3*, and *HDT4*, respectively; Dangl et al., 2001; Pandey et al., 2002; Zhou et al., 2004). Bioinformatics analysis of the HDAC domains of these HD2 proteins revealed a series of highly conserved motifs. A phylogenetic analysis of the nucleotide sequences indicates that a single *HD2* gene led to the development of all *HD2* genes in both monocot and dicot plants (Pandey et al., 2002).

It was demonstrated that *AtHD2A, AtHD2B*, and *AtHD2C* were able to mediate transcriptional repression (Wu et al., 2000, 2003; Zhou et al., 2004). The findings that AtHD2A was required for deacetylation and subsequent methylation of H3 Lys9 supported the idea that HD2 proteins repressed gene expression by modifying histone (Lawrence et al., 2004). Research showed that HD2A, HD2B, and HD2C were all localized in the nucleolus (Lusser et al., 1997; Lawrence et al., 2004; Zhou et al., 2004) and were expressed globally in *Arabidopsis* (Lawrence et al., 2004; Zhou et al., 2004; Hollender and Liu, 2008). Gene expression mediated by *HD2A, 2B*, and *2C* can regulate several aspects of plant development. It was shown that the repression of *AtHD2A* expression led to the development of a short silique and the abortion of seeds (Lagacé et al., 2003). Ectopic expression of *AtHD2A* created a mutant phenotype, characteristics of which included the narrowing and curling of leaves, abnormal flower development, and delayed blooms (Lagacé et al., 2003). *AtHD2B* expression was up-regulated by low temperature and after-ripening (dry storage of mature seed) treatments that are known to break seed dormancy, so suppression of *AtHD2B* expression was important in maintaining seed dormancy (Yano et al., 2013). In tobacco (*Nicotiana tobacum*), *NtHD2a* and *NtHD2b* were found to act as negative regulators of cell death induced by the elicitor cryptogein (Bourque et al., 2011). During longan (*Dimocarpus longan*) fruit senescence under different storage conditions, a histone deacetylase 2-like gene (DlHD2) may interact with an ethylene-responsive factor-like gene (DlERF1) to regulate expression of genes involved in fruit senescence (Kuang et al., 2012).

Several studies have shown that certain HD2s are involved in plant responses to abiotic stresses. Sridha and Wu (2006) showed that expression of AtHD2C was largely repressed by abscisic acid (ABA), a hormone that regulates plant response to abiotic stresses. Over-expression of *AtHD2C* in *Arabidopsis* resulted in plants that were insensitive to ABA. Further, transgenic *AtHD2C* plants exhibited reduced transpiration and increased tolerance to salt and drought stresses compared to wild-type plants. Luo et al. (2012a) showed that several *hd2c* mutants were more sensitive to ABA and NaCl during seed germination. The plants with *HD2C* insertion mutation displayed decreased tolerance to salt stress. Using both BiFC and co-immuno precipitation assays, the study found that HD2C physically interacted with HDA6, another type of HDAC, and the abiotic stress response was modulated through the HD2C/HDA6 complex. Further, research showed that histone H3 was a target of the HD2C/HDA6 complex. A more recent study showed that there existed at least two HD2 genes in rice, *HDT701* and *HDT702*. Overexpression of *HDT701* in rice resulted in an increased tolerance to salt and drought during seedling stage (Zhao et al., 2015). These studies suggest that plant responses to abiotic stresses are controlled by an antagonistic mechanism of histone acetylation and deacetylation on expression of target genes and that HD2s play unique roles in plant reaction to environmental stresses.

Previous research indicates that the expression profile of *HD2D* gene was largely different from other *HD2* genes. The expression *HD2A, HD2B*, and *HD2C* was detected in ovules, embryos, shoot apical meristems, and primary leaves and the expression of these genes were strongly induced during somatic embryogenesis. On the other hand, *HD2D* transcript was only detected in the stems and flowers with young siliques (Zhou et al., 2004). The authors suggest that HD2D may have adopted different functions. At this time, little is known about the specific expression and function of the *HD2D* gene in plants. In this study, we provided evidence that *Arabidopsis* AtHD2D is localized in the nucleus and it is involved in regulating the growth of roots and flowers in *Arabidopsis*. When *AtHD2D* was overexpressed, *Arabidopsis* plants displayed more tolerance to drought, salt, and cold stresses compared to wild-type plants. The research indicates that HD2D is an important protein in plants for environmental stress response.

## Materials and methods

### Gene and protein sequence analyses

The *AtHD2* gene sequence analysis was conducted using the ExPASy net (Bioinfor- matics Resource Portal (http://www.expasy.ch/tools/#proteome). DNAMAN (Version 5.5.2, Lynnon Biosoft) was used forsequence alignment analysis of *AtHD2* coding-sequence (CDS). Considering HD2s belong to one family and share certain similarities, we used Maximum Parsimony method for phylogenetic tree construction. Bootstrap was used to test the developed trees. MEGA 6.0 was used in the analysis. The bioinformation of *AtHD2D* gene expression product was predicted by primary structure analysis. Two software programs, ProtParam, and PSORT II Prediction, were used to predict the subcellular localization of AtHD2 proteins. In addition, the tool for transmembrane regions and signal peptide sequence analysis, ProtComp 9.0 tool (http://linux1.softberry.com/berry.phtml?topic=protcomppl& group=programs&subgroup=proloc) fuzzy k-nearest neighbors (k-NN) algorithm (version 41.0), and TMHMM Server v. 2.0 were used for AtHD2 protein analysis.

### Plant materials and growth

*Arabidopsis thaliana* (ecotype, Columbia) was used in this research. *Arabidopsis* seeds were surface-sterilized in 70% ethanol for 1, 10 min in 10% v/v sodium hypochlorite, and rinsed 5 times with sterile distilled water. The seeds were sown on MS medium (Murashige and Skoog, 1962; Sigma-Aldrich, Lenexa, KS, USA) in Petri dishes. The plants were maintained in a tissue culture room with 16 h of light at 25°C/8 h of dark conditions at 22°C. Germinated seedlings were transferred into soil (PRO-MIX “BX” with Mycorise PRO, Primer Horticulture, Grower Services Canada). Plant seedlings were grown in a growth chamber under 16 h of light at 25°Cand 8 h of dark conditions at 22°C (unless for stress treatments which are described specifically). Each overexpression independent line of *Arabidopsis* was grown in one of conjoined pots (9 × 12 cm, height × diameter). Each pot was planted with 12 seedlings, all from the same clone. The overexpression seedlings of the same independent line were planted into pots randomly for different stress treatments. After plants were transferred into soil, each pot was covered with transparent film for 4 days to avoid rapid water loss.

### DNA constructs

The putative *HD2D* mRNA complete coding sequence (CDS) was obtained from the NCBI (National Center for Biotechnology Information; http://www.ncbi.nlm.nih.gov/nucleotide/145360405?report=genbanklog=nucltop) and TAIR (The *Arabidopsis* Inform- ation Resource (http://www.arabidopsis.org/servlets/TairObject?type=locus&name=AT2G27840) databases. According to the coding sequence, the *35S*:*AtHD2D* vector for overexpression and *35S:AtHD2D-gfp v*ector for HD2D subcellular location were constructed. The *AtHD2D* was amplified using the primer pair of pr1 (5′-ACCAGATCTATGGAG TTTTGGGGTATCGAG-3′) and pr2 (5′- CCACTAGTCTACTTT TTGCAAGAGGGACCAC-3′) and fusion *AtHD2D*-*gfp* was amplified using the pr1 and pr3 (5′-CCACTAGTCTTTTTGC AAGAGGGACCAC-3′; Supplementary Table 1) and all with appropriate restriction sequences (*Bgl*II and *Spe*I). Phusion high-fidelity DNA polymerase (F-530S, 100U New England Biolabs, Inc. USA) was used in the PCR reaction to minimize undesired mutations in the sequences. The construct for subcellular location of AtHD2D was made by in-frame fusion of complete *AtHD2D* CDS with the *gfp* sequence in which the stop codon (TAG) of *AtHD2D* CDS was deleted. The resulting PCR products were cloned to pGEM®-T and pGEM®-T Easy Vector Systems (A1360 Promega Corporation) digested by *Bgl*II and *Spe*I and then transferred into the pCAMBIA1302 binary vector containing the *hptII* coding for hygromycin resistance which was used as the selectable marker for plant transformation selection. The constructs were introduced into *Agrobacterium* strain GV3101/pMP90 by electroporation using the GENE PULSER II system (Bio-Rad, Hercules, CA, USA).

### Plant transformation

The *35S*:*AtHD2D* vector and *35S:AtHD2D-gfp v*ector were introduced into *Arabidopsis* separately for overexpression studies and for subcellular location studies, respectively. *Arabidopsis* transformation followed the floral dip method (Clough and Bent, 1998). T1 seeds were harvested, dried at 25°Cand germinated on MS medium containing 30 μg mL^−1^ hygromycin to select transgenic seedlings, and 50 μg mL^−1^ carbenicillin was added to prevent *Agrobacterium* contamination. Seeds obtained from the primary transgenic lines were germinated on antibiotic–containing medium, and PCR analyses were performed on resistant plants. Surviving and PCR analysis positive T1 plantlets were transferred to soil to harvest T2 seeds. T2 seeds were germinated as described before and T3 seeds were harvested. Plants developed from T3 seeds were used as the materials for all experiments.

To produce the *Arabidopsis* plants for confocal fluorescence observation, seeds were sown on MS medium in vertical plates and the plants were grown for 6 days. Root tips were harvested and used for observation. *Nicotiana benthamiana* were grown as described by Han et al. (2013), and plants were maintained in growth chambers for investigating the subcellular localization of HD2D-GFP in transient gene expression experiments. The chamber conditions were set at 14 h light and 10 h dark at 21°C. Young leaves were infiltrated with *Agrobacterium* GV3101/pMP90 containing the *HD2D-gfp* construct and observations were conducted 48–72 h after infiltration.

### Molecular analysis of plants

Total genomic DNA was extracted from plant leaves using a CTAB method as described by Wang et al. (1993). Total RNA was isolated from leaves using TRIzol reagent (Invitrogen Life Technologies, Carlsbad, CA) following manufacturer's instructions. Primers specific to the *hptII* gene for a 563 bp PCR product (Supplementary Table 1) were used in PCR analysis of plants. The pr1 and pr2 primers specific for *HD2D*, and the pr1 and pr3 primers specific for *HD2D-gfp* were also used in PCR analysis. The primer for the *Actin2* gene of *Arabidopsis* (Czechowski et al., 2004) was used as an internal control.

All PCR reactions were performed with 1 unit of Taq polymerase (GenScript, the Biology Co, USA), 0.2 mM dNTPs, and a pair of primers (0.1 μM each) in a final volume of 20 μl. PCR was conducted at 94°C for 2 min followed by 30 cycles of 94°C for 15 s, 55–57°C for 20 s, and 72°C for 45 s, with a final polymerization step at 72°C for 3 min.

For RT-PCR analysis, 2 μg of each total RNA sample was treated with the RNase-free DNase according to manufacturer's instructions (Promega). Treated RNA samples were desalted (to prevent carry-over of magnesium) before cDNA synthesis using Microcon-100 spin columns (Millipore). The first-strand cDNA was synthesized using random hexamers and SuperscriptII reverse transcriptase (Invitrogen), according to manufacturer's instructions, and subsequently diluted with nuclease-free water (Sigma) to 12.5 ng μl^−1^ cDNA. Reactions were run on a thermocycler (model Mastercycler gradient; Eppendorf Canada Ltd., Mississauga, Ontario) using the same conditions as described above.

### Plant growth evaluation

Only seed batches that had been harvested and stored at the same time and under the same conditions were used. Seeds were sown on MS medium, vernalized at 4–8°C and then transferred to a tissue culture room under 16 h of light at 25°C/8 h of dark at 22°C. The germination rate (defined as the emergence of the hypocotyl axis through the seed coat) was determined at different days after seeds were placed for germination. Experiments were conducted in triplicate with 180 seeds for each experiment. For investigation of root growth, seeds were sterilized and grown on vertically oriented MS agar plates in a tissue culture room. Transgenic plants and wild-type plants were grown in adjacent parallel rows in the same Petri dishes (9 cm), 10 transgenic seedlings in one row and 10 wild-type seedlings in the other. After photos were taken, the number of lateral roots was counted under a dissecting microscope. The plants were placed on a flat glass and then main roots and lateral roots were straightened as much as possible using a forceps. A verniercalliper was used for measuring the length of main and lateral roots. For determination of root and shoot ratio, 10 day old plants were removed from plates, medium residues were cleaned from plants and plants were blotted dry using filter papers. Roots were cut off from stems (with leaves) and roots and shoots were dried in an oven. The dry matters were weighed and root:shoot ratio was calculated. For flowering assessment, 5 days after seed germination on MS medium, the seedlings were transferred into soil-containing pots. Twenty-six days after germination when plants started to show the rosette stage of leaf growth and plants showed sign of flowering, flowering was evaluated, which was based on plant bolting and the formation of flower buds. All tests were replicated three times and each replicate consisted of at least six independent lines.

### Stress treatment

Stress treatments were initiated after plants were grown for 10–13 days in soil under regular conditions. Each treatment included six independent lines with three replicates for overexpression and wild type. The results presented in figures were calculated based on the values of all six independent lines and three replicates. The drought treatment was conducted by withholding water after seedlings were transplanted into pots. For cold treatment, plants were moved to a growth chamber at 2°C, and leaf samples were collected every 2 days. For salt stress, water containing 300 mmol per L of NaCl was evenly spayed on the soil surface and leaf samples were collected every 3 days.

With the time extension under stresses, plant leaves gradually turned brown then dark brown. When 50% leaf area had turned brown, the leaf was counted as brown leaves. Leaves under salt stress gradually turned yellow and wilted. When 50% leaf area had turned yellow the leaf was recorded as withered leaves.

### Detection of GFP signals by confocal laser scanning microscopy

Transient expression and stable expression of 35S:GFP and 35S:AtHD2D-GFP fusion protein in *N. benthamiana* leaves and *Arabidopsis* root tips were detected using laser fluorescence microscopy (Sparkes et al., 2006). Photographs were taken using a confocal florescent microscope (Leica Microsystems, Germany) under excitation at 450–490 nm, emission at 520 nm and dichroic mirror at 510 nm.

### Malondialdehyde (MDA) and electrolyte leakage and analysis

Malondialdehyde (MDA) content and electrolyte leakage were used to analyze stress damage to plants (Campos and Nunes, 2003). For MDA analysis, the cell injury level was expressed as the content of MDA according to Zhang et al. (2005). Fresh leaves from each treatment were homogenized in 5 mL of 10% trichloroacetic acid (TCA), and then centrifuged at 4000x g for 20 min. To each 2 mL aliquot of the supernatant, 2 mL of 0.6% thiobarbituric acid in 10% TCA was added. The mixtures were heated at 100°C for 15 min and then quickly cooled in an ice bath. After centrifugation at 10,000x g for 20 min, the absorbance of the supernatant was recorded at 532 and 450 nm. Lipid peroxidation was expressed as the MDA content in nmol per g FW. For electrolyte leakage analysis, freshly cut leaf discs of 0.5 cm^2^ each were rinsed 3 times (2–3 min) with demineralized water and subsequently floated on 20 mL of demineralized water. The electrolyte leakage in the solution was measured after 24 h room temperature using a conductivity meter (Smart Stability, Orion, Thermo Scientific U.S.A). Total conductivity was obtained after the flasks were maintained in an oven (90°C) for 2 h.

### Statistical analysis

Data analysis was performed using SPSS 16.0.2 computer statistical program (IBM SPSS, 2008). The data are presented as the mean ± standard deviation of the mean (*SD*) and were tested for significant difference using Student's *t*-test. *p* < 0.05 was selected as the point of minimal significant difference in all of the analyses.

## Results

### Bioinformatics analyses of *AtHD2* genes and proteins

**Figure 1:**
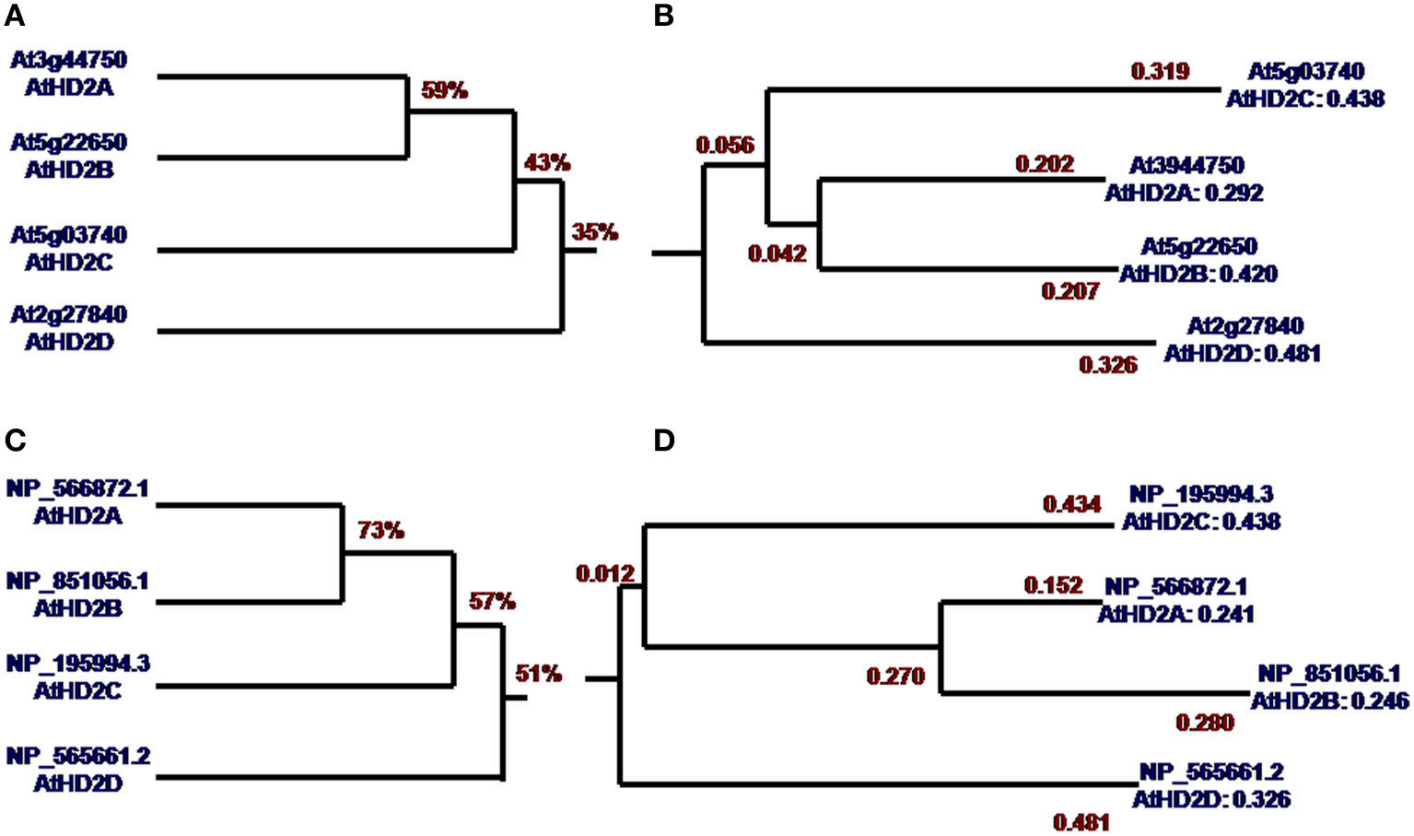
**Homology tree and phylogenetic tree of *AtHD2* genes and AtHD2 proteins. (A)** CDS of *AtHD2* gene homology tree. **(B)** CDS of *AtHD2* gene phylogenetic tree. **(C)** AtHD2 protein homology tree. **(D)** AtHD2 protein phylogenetic tree.

DNA sequence analysis showed that the overall homology of *AtHD2* genes is 35% (Figure 1A). Sequence homology of *AtHD2A* and *AtHD2B* was 59% which is the highest. *AtHD2D* is distantly related to the other *AtHD2* genes, and this observation is supported by the sequence homology value of only 35%. Meanwhile the phylogenetic tree showed that the genetic distance (GD) between *AtHD2A* and *AtHD2B* was 0.042, next was *AtHD2C*, and the largest GD was *AtHD2D* with a value of 0.056 (Figure 1B). Protein sequence alignment showed that the overall similarity of the HD2 proteins was 51% (Figure 1C). The phylogenetic tree showed that AtHD2A and AtHD2B were the closest, the GD being the smallest. On the other hand, the AtHD2D protein was furthest among the HD2 proteins with a GD-value of 0.481 (Figure 1D).

The analysis of *HD2* genes showed that *HD2D* is largely different from other *HD2* genes. The molecular weight of *HD2D* is significantly lower compared to other *HD2* genes (Supplementary Table 2). While *HD2A, HD2C* genes contain the zinc finger domain, the *HD2D* lacks this important region. The *HD2D* showed the lowest random coil value and the highest values of β-turn, pI and extended strand (Supplementary Table 2).

### Plant transformation and transgene gene expression analyses

**Figure 2:**
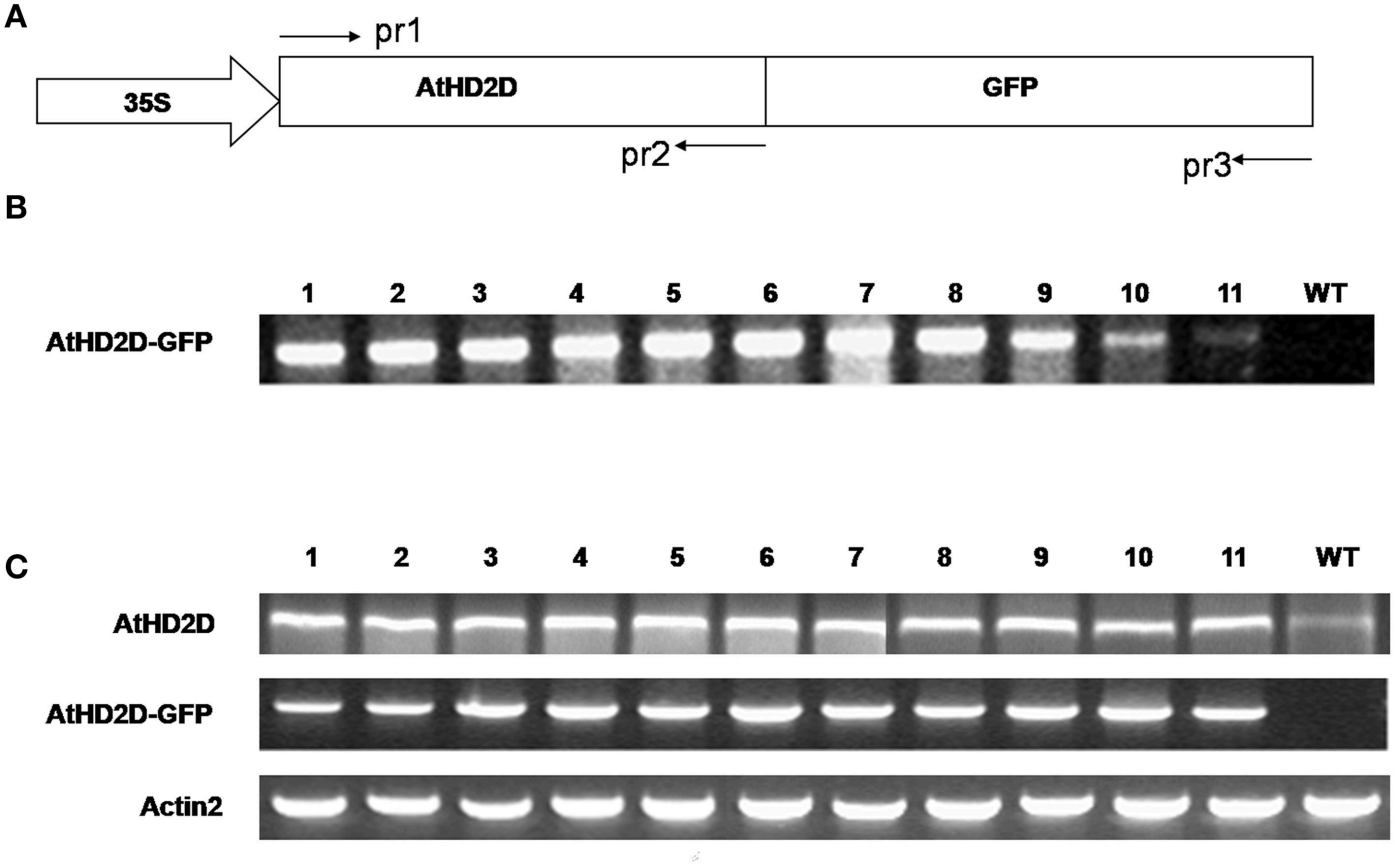
**Expression analysis of *AtHD2D* and of *AtHD2D-gfp* in transgenic *Arabidopsis thaliana* plants. (A)** Diagram of the *35S:AtHD2C-gfp* construct. *AtHD2D* CDS was fused with the *gfp*-coding region driven by a 35S promoter. The primers, pr1, pr2, and pr3, used to analyze *AtHD2D* and *AtHD2D-gfp* in transgenic plants were shown. **(B)** PCR analysis of *35S:AtHD2D-gfp* transgenic and wild-type (WT) plants using primer pairs of pr1 and pr3. No PCR product was found in WT. **(C)** RT-PCR analysis of *AtHD2D* and *AtHD2D-gfp* expression in transgenic and wild-type plants using the primer pairs of pr1 and pr2 or pr1 and pr3. No RT-PCR product of *AtHD2D-gfp* was found in WT.

Plants of 30 randomly selected independently lines developed on medium containing hygromycin were analyzed by PCR and RT- PCR using specific primers (Supplementary Table 1, Figure 2A). All the lines analyzed were PCR positive for *HD2D* using pr1 and pr2 primer pair as shown in a representative image Figure 2B. The RNA expression of transgene was analyzed for randomly selected independent lines using RT-PCR using transgene specific primers (Figures 2A,C, Supplementary Table 1) and the *actin2* gene was used as the internal control. The RNA expression of *AtHD2D* was increased in *35S:AtHD2D* transgenic lines comparing to the control (Figure 2C). Plants transformed with *AtHD2D*-*gfp* showed expression of the fused gene (Figure 2C) but no expression of *AtHD2D-GFP* was observed in the non-transformed wild type. Expression of *actin2* as the internal control was consistent in both non-transformed wild types and transformed plants (Figure 2C). These results confirmed the expression of transgenes in plants developed following transformation.

### Expression localization of *HD2D*

**Figure 3:**
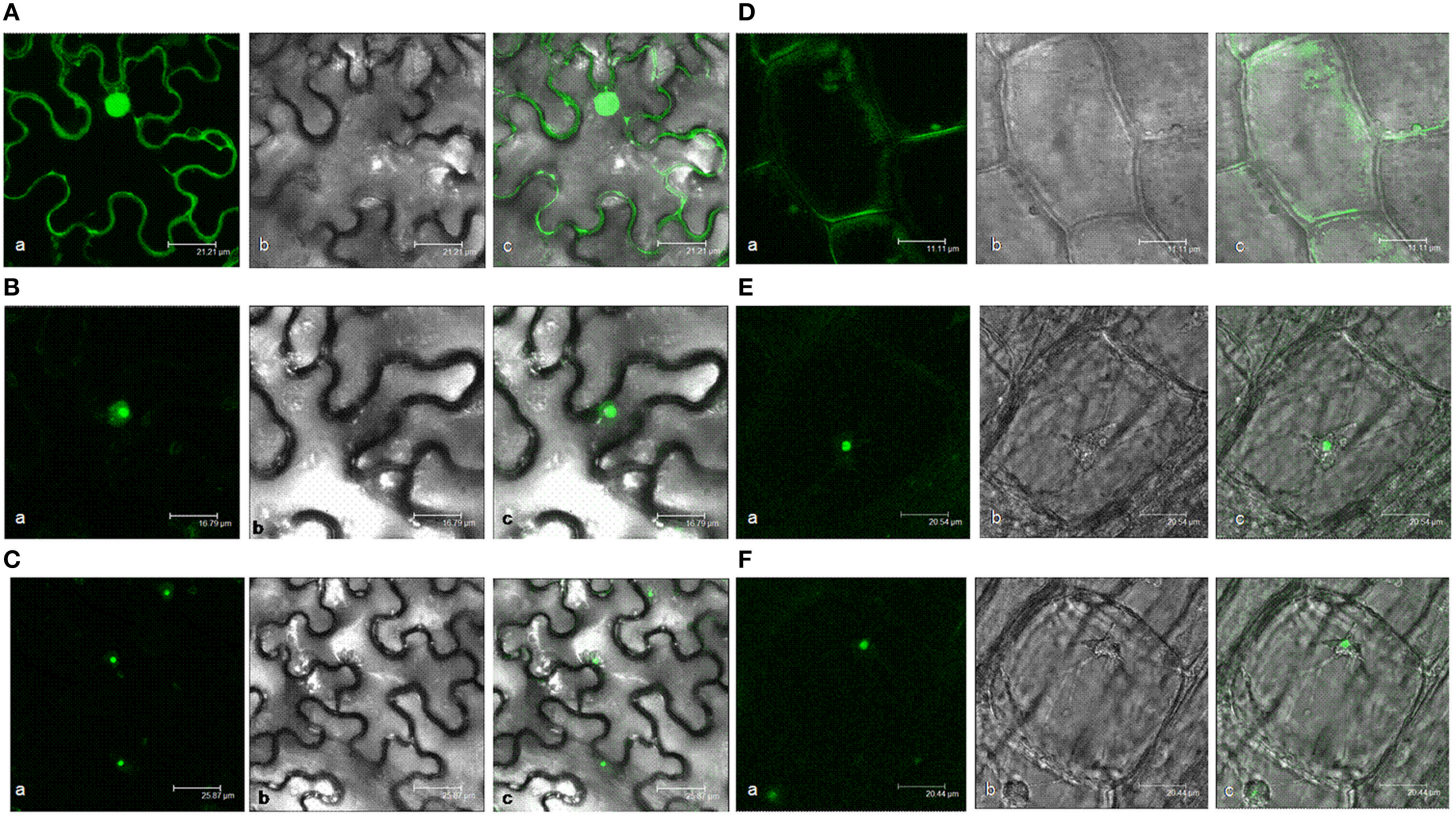
**Subcellular localization analyses of 35S:AtHD2D and 35S:AtHD2D-GFP**. (a) Fluorescence image, (b) With white light, (c) Merge of fluorescence and bright images. **(A)** Subcellular localization of 35S:GFP in *N. bentamiana*. **(B)** Subcellular localization of AtHD2D-GFP in *N. bentamiana*. **(C)** General view of subcellular localization of AtHD2D-GFP in *N. bentamiana*. **(D)** Subcellular localization of 35S:GFP in root tip of transgenic *Arabidopsis*. **(E)** Subcellular localization of 35S:AtHD2D-GFP in root tip of transgenic *Arabidopsis*. **(F)** General view of subcellular localization of 35S:AtHD2D-GFP in root tip of transgenic *Arabidopsis*.

Two software programs, ProtComp and PSORT II Prediction, were used to predict the subcellular location of AtHD2 protein. The HD2D protein was positioned in the cell nucleus but with the lowest value compared to other *HD2* genes (Supplementary Table 3) and it does not have a membrane spanning domain nor a signal peptide. Meanwhile, HD2D showed a high possibility to localize in cytoplasm and the highest possibility to be distributed in cytoskeleton, mitochondria, and peroxisome. AtHD2D also had the lowest integral prediction value. The actual location of the AtHD2D protein was studied via gene expression experiments. *N. benthamiana* leaves were infiltrated with a construct containing *AtHD2D* fused with *gfp* and the expression was observed 48–72 h after the infiltration. The *gfp* gene alone driven by CaM35S promoter showed constitutive protein expression activity in plant cells (Figure 3A). The expression of AtHD2D-GFP was mainly localized in nucleus (Figures 3B,C). There were halos around the fusion protein, as opposed to clear boundariess which were very sharp and without a fringe or halo, as seen in other AtHD2 proteins.

AtHD2D subcellular localization was further studied via stable expression of the gene in *Arabidopsis*. Two transgenic lines with higher levels of *AtHD2D-gfp* expression were selected in the study. Stable expression of *AtHD2D* gene in transgenic *Arabidopsis* root cells was observed. Plants transformed with *gfp* driven by 35S promoter showed constitutive expression in root cells (Figure 3D). Plants transformed with a construct carrying the fusion *AtHD2D-gfp* showed that GFP was expressed in the nucleus (Figures 3E,F). Both transient and stable expression studies indicated that the location of AtHD2D is mainly in the cell nucleus.

### Involvement of AtHD2D in plant growth

**Figure 4:**
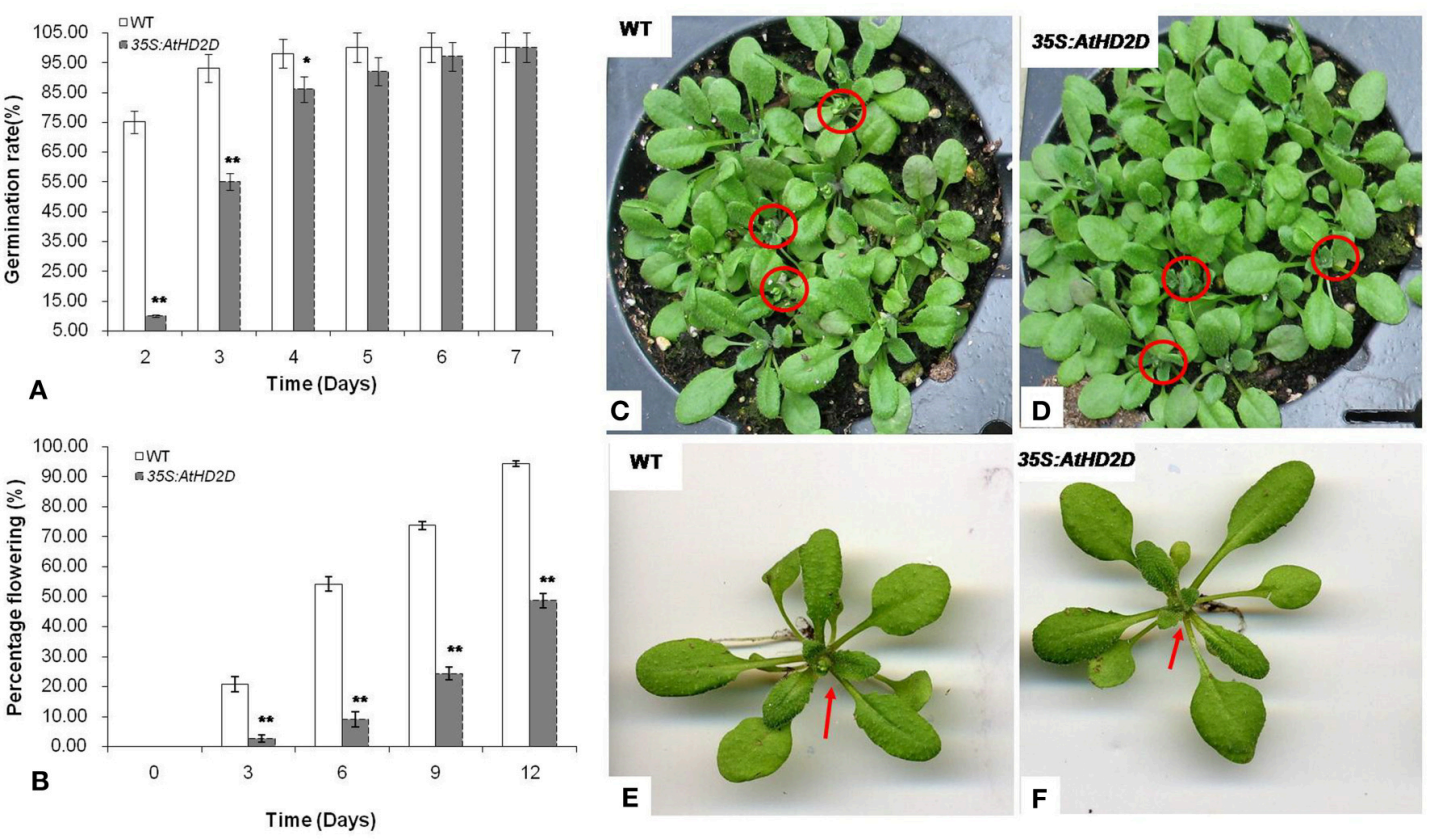
**Seed germination and flowering of wild type (WT) and *35S: AtHD2D* transgenic plants under regular conditions on MS medium**. The data are presented as the mean ± standard deviation of the mean (*SD*) and were tested for significant difference using Student's *t*-test (^*^p < 0.05, ^**^p < 0.01). **(A)** Seed germination rate of WT and *35S:AtHD2D* transgenic plants. **(B)** Plant flowering rate of WT and *35S:AtHD2D* transgenic seedlings. **(C,E)** WT seedlings. **(D,F)** *35S:AtHD2D* transgenic seedlings.

Seed germination was evaluated for plants overexpressing the AtHD2D. Germination of seeds from transgenic plants was slower compared to the wild-type (Figure 4A). While germination of the wild type seeds was 75.6% in 2 days and 98.2% by day 4, germination of transgenic seeds was delayed by 5–6 days.

Flowering time was significantly delayed in transgenic plants (Figure 4B). During the 12 day observation period, transgenic plants consistently showed lower flowering rates compared to wild type plants. By day 12, about 90% of WT plants showed bloom development, but transgenic plants were largely still in a vegetative growth stage (Figures 4C–F).

**Figure 5:**
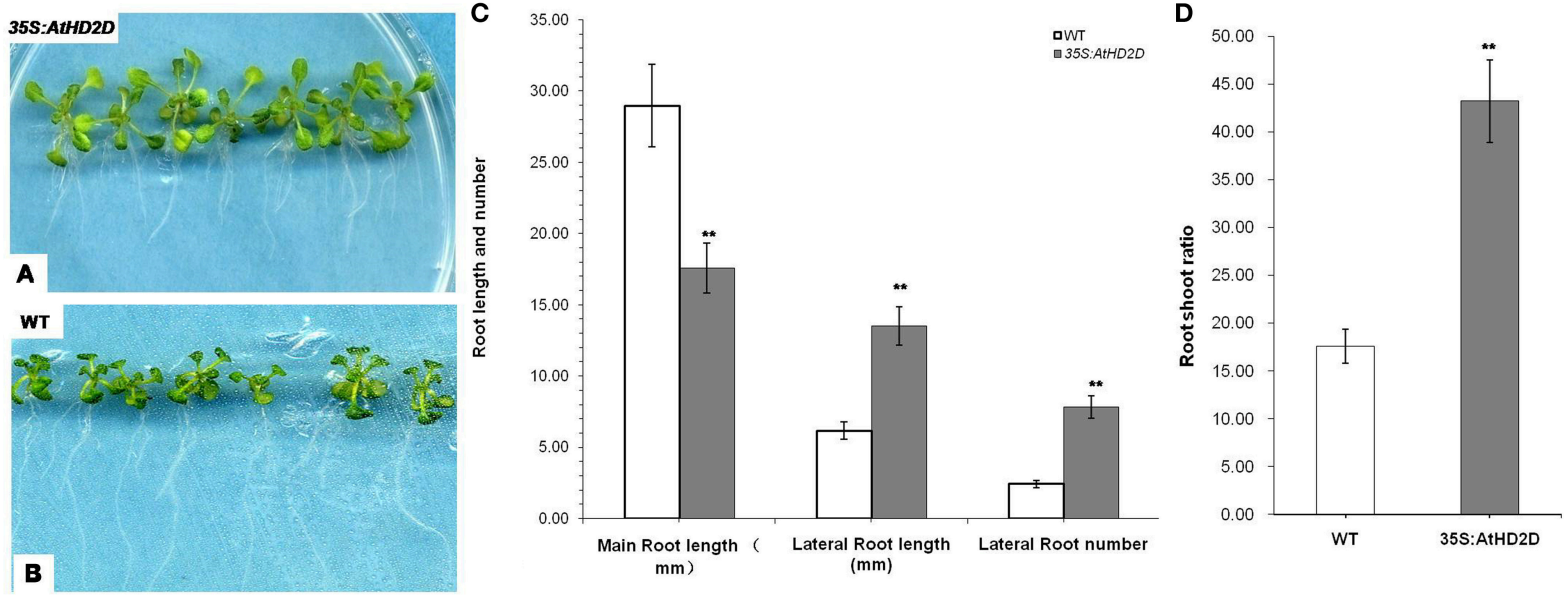
**Root development of wild-type and *35S:AtHD2D* transgenic plants**. The error bars represent the *SD*. The data are presented as the mean ± standard deviation of the mean (*SD*) and were tested for significant difference using Student's *t*-test (^**^p < 0.01). **(A,C)** Roots of wild-type (WT) 10 days after germination. **(B,D)** Phenotype of roots and leaves of *35S:AtHD2D* of transgenic seedlings 10 days after germination. **(E)** Root and shoot ratio of WT and *35S:AtHD2D* transgenic plants 10 days after germination on MS medium. **(F)** Root length and root number of WT and *35S:AtHD2D* transgenic plants.

Ten days after germination on MS medium, the root development was evaluated. The plants overexpressing AtHD2D had shorter main roots and longer lateral roots (Figures 5A,C). In contrast, the wild type plants under the same growth conditions had obviously longer main roots but shorter lateral roots (Figures 5B–D). Transgenic plants also had more lateral roots compared to wild type plants (Figures 5A,C). The root:shoot ratio of transgenic plants was higher than that of wild type plants (Figure 5D).

### Involvement of HD2D for stress tolerance

**Figure 6:**
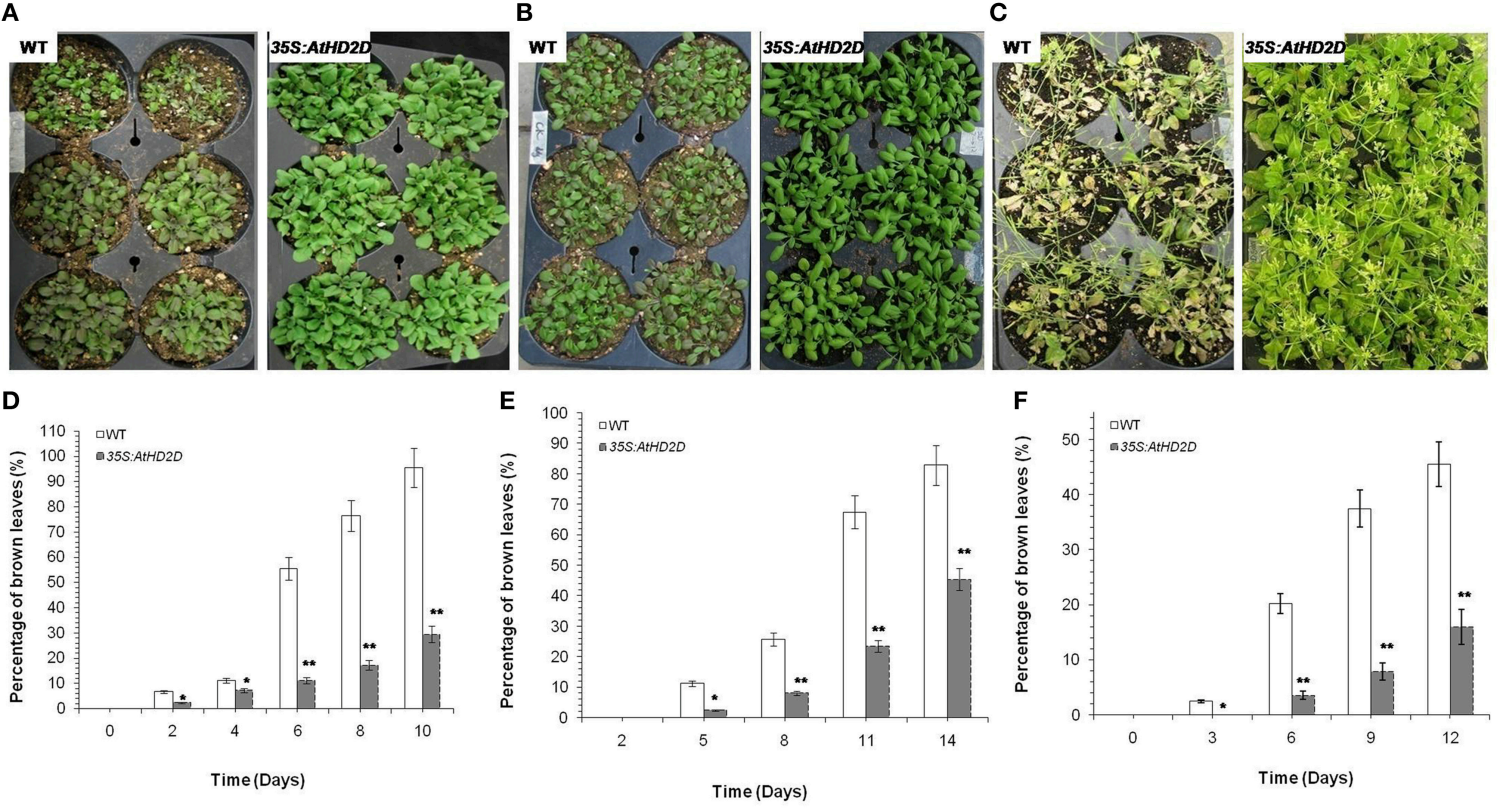
**Full view of wild type (WT) and *35S:AtHD2D* transgenic plants under abiotic stresses**. The error bars represent the *SD*. The data are presented as the mean ± standard deviation of the mean (*SD*) and were tested for significant difference using Student's *t*-test (^*^p < 0.05, ^**^p < 0.01). **(A)** WT and 35S:AtHD2D transgenic plants under cold stress for 8 days. **(B)** WT and *35S:AtHD2D* transgenic plants under drought stress conditions for 9 days. **(C)** WT and *35S:AtHD2D* transgenic plants under salt stress conditions for 18 days. **(D)** Browning levels in leaves of WT and *35S:AtHD2D* transgenic plants under cold stress. **(E)** Browning levels in leaves of WT and *35S:AtHD2D* transgenic seedlings under drought stress. **(F)** Browning levels in leaves ofWT and *35S:AtHD2D* transgenic seedlings under salt stress.

Plants overexpressing AtHD2D showed increased tolerance to cold, drought, and salt stresses (Figure 6). Under cold and drought conditions, the *HD2D* overexpressing plants grew normally and retained normal green color in leaves (Figures 6A,B,D,E). However, wild type plants grew slowly and the leaves wilted and turned brown and showed stressed phenotypes. Under salt stress, wild type plants became yellow in color and plants were obviously injured but plants overexpressing HD2D grew relatively normally and produced flowers (Figures 6C,F).

**Figure 7:**
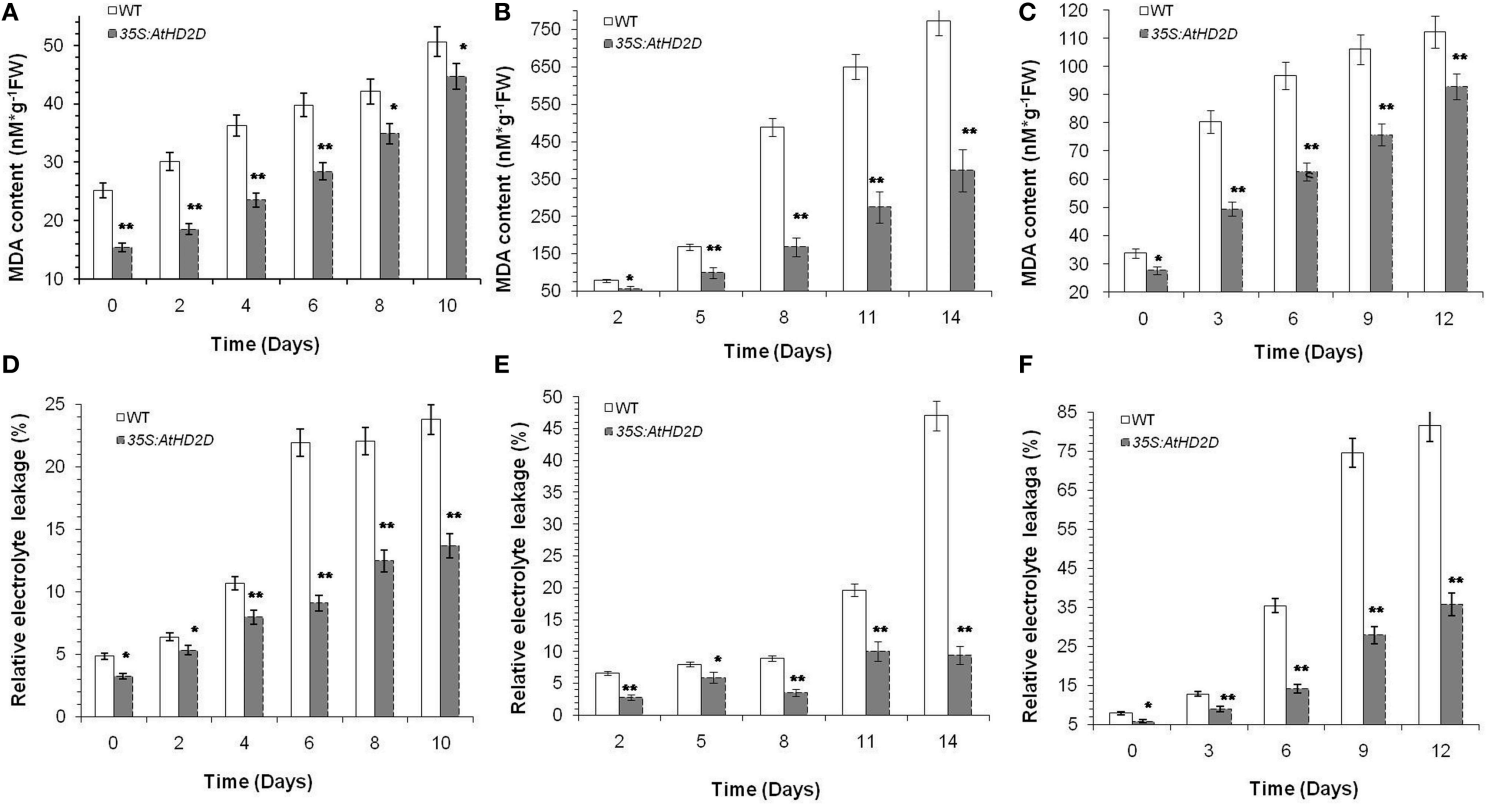
**Malonaldehyde (MDA) and electrolyte leakage levels in WT and *35S:AtHD2D* transgenic plants under abiotic stresses**. The error bars represent the *SD*. The data are presented as the mean ± standard deviation of the mean (*SD*) and were tested for significant difference using Student's *t*-test (^*^p < 0.05, ^**^p < 0.01). **(A)** MDA level under cold stress condition. **(B)** MDA level under drought stress condition. **(C)** MDA level under salt stress condition. **(D)** Electrolyte leakage level under cold stress. **(E)** Electrolyte leakage level under drought stress. **(F)** Electrolyte leakage level under salt stress.

Based on Campos and Nunes (2003), MDA and electrolyte leakage were conducted. MDA levels in transgenic plants overexpressing AtHD2D increased slowly under cold, drought and salt conditions, while MDA levels in wild type leaves increased rapidly over the time (Figures 7A–C). Analysis of electrolyte leakage from leaves (an indicator of cell membrane damage) during the course of abiotic stresses showed that electrolyte leakage of wild type plants increased steadily, while electrolyte leakage in transgenic plants overexpressing AtHD2D increased slowly and remained relatively stable (Figures 7D–F).

## Discussion

HDACs function by removing the acetyl groups from lysine residues, thus allowing the nucleosomes to pack more tightly which consequently represses transcription (Song et al., 2005; Liu et al., 2014). Silencing gene expression by HDACs is selective and altered gene expression controls certain biological processes, especially plant development and response to environmental stresses (Kim et al., 2010; Yano et al., 2013; Kim et al., 2015; Pi et al., 2015; Yang et al., 2015; Zhao et al., 2015). Even in the earliest phases of plant development, such as embryogenesis, the histone code is necessary to establish the correct developmental pathways (Tai et al., 2005; Köhler and Makarevich, 2006; Long et al., 2006; Kim et al., 2010; Wickramasuriya and Dunwell, 2015). During later stages of the plant life cycle, histone modifications influence growth phase change (Vlachonasios et al., 2003; Chua et al., 2005; Rossi et al., 2007; Yano et al., 2013), flowering timing (He and Amasino, 2005; Deng et al., 2007; Luo et al., 2015), and maternal imprinting and fertilization (Guitton and Berger, 2005; Köhler and Makarevich, 2006).

The first *HD2* was discovered in maize (Lusser et al., 1997). A comparative analysis of the HD2 type of HDAC proteins in higher plants revealed three *HD2* genes in maize and four genes in *Arabidopsis* (Dangl et al., 2001). The uniqueness and specificity of HD2s to plants suggests this type of HDACs possess specific features and functions (Grandperret et al., 2014). Previous studies have focused on *HD2A, HD2B*, and *HD2C* and less research has been done for *HD2D*. Limited research indicates, to certain extent, that *HD2D* is different from other *HD2* genes in expression and potential functions (Zhou et al., 2004). Our research studied *AtHD2D*, especially with respect to plant development and plant response to environmental stresses.

Bioinformatics analysis and prediction are based on the information accumulated in molecular biology databases. We did a series of bioinformatics analysis on *AtHD2D*. The DNA sequence analysis showed that *AtHD2D* tends to be distantly related to other *AtHD2* genes. The phylogenetic tree also shows the genetic distance of *AtHD2D* is the furthest. Protein sequence alignment also showed that AtHD2D protein is less related to other HD2 proteins. The analysis of *AtHD2* gene composition shows that *AtHD2D* appears to be different from other *AtHD2* genes in several categories. All these suggest that *AtHD2D* is relatively unique among *AtHD2* genes. Although it retains certain characteristics typical of *AtHD2* genes, *AtHD2D* probably branched off from *AtHD2s* a long time ago during evolution.

AtHD2s have a conserved N-terminal EFWG (well-conserved motif) amino acid region which is required for repression of gene expression, followed by a central acidic region rich in glutamic and/or aspartic acids. In AtHD2A, this acidic region is essential for catalytic activity, since the deletion of this region resulted in a compromised HDAC activity (Wu et al., 2000). HD2A and HD2C contain a single C2H2 type zinc finger domain in the carboxyl terminus, which may enable high affinity DNA-binding or mediate protein-protein interactions (Aravind and Koonin, 1998; Wu et al., 2000; Dangl et al., 2001; Zhou et al., 2004). Previous research showed that while other *AtHD2A, AtHD2B*, and *AtHD2C* exhibited an effective repression of gene expression, *AtHD2D* was much weaker to repress reporter gene expression (Zhou et al., 2004). As AtHD2B also lacks the zinc finger domain, the zinc finger may not be essential for repression of gene expression.

The analysis revealed that there is no signal peptide in the full coding sequence of AtHD2D, therefore, HD2D is not a secretory or mosaic protein in membranes. Bioinformatics analysis indicated that the products of *AtHD2D* tend to be in cytoplasm. However, transient expression analysis in *N. benthamiana* and stable expression in *Arabidopsis* suggested that AtHD2D-GFP was mainly localized in the nucleus of cells in both plant species. A study by Luo et al. (2012b) showed HD2D interacted with RPD3 family proteins of HDA6 and HDA19 and the signals were observed in cell nucleus, suggesting nucleus localization of HD2D. AtHD2D was not diffusely distributed in the nucleoplasm but was localized in approximately half the nucleus. The results also indicate that sequence analysis predictions do not reflect the real expression and localization of AtHD2D in cells and these outcomes are different from other AtHD2 proteins. A recent study by Zhao et al. (2015) showed that different members of tomato SiHDACs had different subcellular localizations and they proposed that different *SiHDAC* genes have distinct functions. Bioinformatics analysis and gene expression localization study results suggest that *AtHD2D* is unique among AtHD2s with respect to gene and protein composition as well as the localization of gene expression (Lawrence et al., 2004; Zhou et al., 2004; Sridha and Wu, 2006). The differences in these categories suggest the *HD2D* may have some different functions among *HD2s*.

Plant phenotypical characteristics of root development showed that *35S-AtHD2D* transgenic plants had more lateral roots compared to wild-type. Root:shoot ratio was also higher in transgenic plants. Nguyen et al. (2013) showed that histone deacetylase inhibitors impeded root development including primary root elongation as well as lateral root emergence. The research further showed that the lateral root growth was significantly reduced in *hda19* seedlings in which the *HDA19* was mutated. More lateral roots and higher root:shoot ratios as shown in this study suggest that such structures can help plants to obtain more nutrients and water, which enable plants to cope with unfavorable conditions, especially environmental stresses. The results indicated that transgenic plants expressing higher levels of *HD2D* may adopt more plasticity in response to abiotic stresses, particularly drought stress.

Histone modification appears to act by integrating signals from different environment stimuli in order to regulate plant growth and development (Stockinger et al., 2001; Lee et al., 2005). Published data also show that each HDAC may have special functions during plant development or upon exposure to certain environmental stimulations, and moreover, one HDAC enzyme can also affect several processes in plant growth (Pi et al., 2015, Kim et al., 2015). The switch from vegetative growth to flowering is a major developmental transition that is initiated by environmental signals, such as the cold weather (vernalization) and changes in day length. Several studies have directly or indirectly indicated involvement of *HDACs* in plant flowering (Sheldon et al., 1999, 2000; Bastow et al., 2004; Sung and Amasino, 2004; He and Amasino, 2005; Deal et al., 2007; Yu et al., 2011; Krogan et al., 2012; Luo et al., 2015). However, not all HDACs are involved in plant development and flowering, and further, the mechanisms by which a HDAC is involved in plant growth and development may be different for different *HDAC* genes (Nguyen et al., 2013; Van Zanten et al., 2014). Our study showed that *AtHD2D* overexpressing plants exhibited delayed seed germination. The flowering time was also delayed in plants overexpressing *AtHD2D*. A recent study by Farhi et al. (unpublished results) showed that a significantly higher number of rosette leaves was observed in *AtHD2D*-overexpressing *Arabidopsis* plants when these plants exhibited delayed flowering (Farhi and Tian, unpublished results). This indicates the late flowering is due to longer vegetative plant growth. Our study demonstrates that *AtHD2D* directly influenced plant development. A longer period of vegetative growth or delayed onset of flowering can help plants to absorb more nutrients for later reproductive growth to cope with environment stresses. Further study is needed to reveal the mechanisms by which HD2D is involved in plant growth and development.

We conducted further research to test AtHD2D responses to abiotic stresses. Transgenic *35S:AtHD2D* plants were grown under drought, cold, or salt stress conditions. *AtHD2D* transgenic seedlings displayed a decreased sensitivity to drought, cold, and salt stresses. Further, changes of MDA and electrolyte leakage were analyzed in *35S*:*AtHD2D* transgenic plants and control plants. Higher content of MDA inhibits cell metabolism and can lead to cell death (Campos and Nunes, 2003). When wild type seedlings were exposed to stresses, MDA content increased quickly. On the other hand, MDA in the *35S:AtHD2D* transgenic seedlings increased slowly and the values remained lower throughout stress treatments. Electrolyte leakage in leaves under stress indicates membrane damage and subsequent cell damage (Rolny et al., 2011), with electrolyte leakage increasing as damage increases. Analysis showed that electrolyte leakage of *35S:AtHD2D* transgenic seedlings were not largely affected under different stresses while the electrolyte leakage in controls increased along with the stress treatments. These results indicate that *AtHD2D* is positively involved in plant response to different environmental stresses.

A recent study showed that *Athd2c* T-DNA insertion mutants (*hd2c-1* and *hd2c-3*) were more sensitive to ABA and salt during seed germination (Luo et al., 2012a). Sridha and Wu (2006) showed that while Arabidopsis overexpressing *AtHD2C* exhibited an increased tolerance to drought and salt stresses, the expression of endogenous *AtHD2C* was decreased in response to ABA treatment. It would be interesting to investigate if involvement of AtHD2D in stress tolerance is regulated by ABA or by other signals. At this time, the number of studies on the relationship of HD2D as well as other HD2s and ABA are very limited. Along with more research in this area, the relationships of HD2D/HD2s with ABA will be elucidated. Although several HDACs have been shown to involve in plant response to stresses, research also indicates individual HDACs play different roles in plant stress reaction and certain HDACs can interact each other as well as interact with other proteins to exert the full function in stress response pathways in plants (Luo et al., 2012a; Perrella et al., 2013). A study by Luo et al. (2012b) using bimolecular fluorescence complementation (BiFC) assay indicated that HD2D interacted with RPD3 family members HDA6 and HDA19. A recent study by Farhi and Tian showed that HD2D interacted with HD2A and HD2C via BiFC assay (Farhi and Tian, unpublished results). These studies suggest that there might exist a protein complex that modulates gene expression for plant development and plant response to stresses. Indeed, plant growth, especially plant growth and development under environmental stress situations should be a complicated process and could need the function of multiple and related proteins, such as HDACs/HD2s. At this time, the studies only demonstrated there were physical interactions among HDAC proteins, research has not shown the function of the protein interactions yet. Further study should aim to elucidate the relationship of HDACs and HD2s in plant development and in plant response to abiotic stresses.

Genome sequencing of many plant species has provided a fundamental base to study the mechanisms underlining plant life at molecular level. Nevertheless, it has become clear that the DNA sequence itself may not be the determining factor in genetic control of plant life. Epigenetic factors are important in regulating plant phenomena. *HD2s* are plant specific epigenetic factors that are heavily involved in a number of plant processes. In our study with *HD2D* gene sequence and protein composition analyses showed that *HD2D* is less related to the other *HD2* genes. *HD2D* is involved in the control of plant development including seed germination, root development and flowering. Our research further showed that *AtHD2D* is also involved in plant responses to environmental stresses, including drought, cold, and salt stimuli. Recent studies have showed several HDAC and HD2 proteins interacted each other in BiFC assay (Luo et al., 2012a,b), indicating certain HADC/HD2s may coordinate to function in response to stress. Further research, especially on the aspect of protein interaction may elucidate the biological mechanisms of HD2D in plants, and that knowledge will be used to manipulate gene expression to increase plant resistance to environmental stresses.

## Author contributions

ZH Conceived and designed the experiments then performed the experiments and analyzed the data, finally wrote the paper. HY Designed the experiments then performed the experiments; analyzed the data, finally wrote the paper. ZZ Conceived and designed the experiments and wrote the paper. DH Conceived and designed the experiments and wrote and revised the paper. XL Performed the experiments and analyzed the data; JD Conceived and designed the experiments. LT Conceived and designed the experiments and wrote and revised the paper.

## Funding

This work was partially supported by China Scholarship Council, National Natural Science Foundation of China (31400233), Program of Basal Scientific Research and Innovation Science, and Northwest A&F University of China (2014YB035)

### Conflict of interest statement

The authors declare that the research was conducted in the absence of any commercial or financial relationships that could be construed as a potential conflict of interest. The reviewer (AM) and Handling Editor declared their shared affiliation, and the Handling Editor states that the process met the standards of a fair and objective review.
