# Supplementary material for: AtHD2D Gene Plays a Role in Plant Growth, Development, and Response to Abiotic Stresses in Arabidopsis thaliana
Source: Front Plant Sci. 2016 Mar 31;7:310. doi: 10.3389/fpls.2016.00310 (PMC4815178; doi:10.3389/fpls.2016.00310)
Supplement: Supplementary file 1 [file Table1.DOC]

**Supplementary Materials**

Additional Supplementary information may be found in the online version of this article

**Table S1 Primers used in the research.**

| Gene’s name | Sequence of primer 5’→3’ | Product size (bp) |
| --- | --- | --- |
| *AtHD2D* | pr1: ACCAGATCTATGGAGTTTTGGGGTATCGAG  pr2:: CCACTAGTCTACTTTTTGCAAGAGGGACCAC | 612 |
| *AtHD2D-GFP* | pr1: ACCAGATCTATGGAGTTTTGGGGTATCGAG  pr3: CCACTAGTCTTTTTGCAAGAGGGACCAC | 1320 |
| *hptII* | hptII F: CAATGACCGCTGTTATGCGG  hptII R: CTCGGAGGGCGAAGAATCTC | 563 |
