# Supplementary material for: AtHD2D Gene Plays a Role in Plant Growth, Development, and Response to Abiotic Stresses in Arabidopsis thaliana
Source: Front Plant Sci. 2016 Mar 31;7:310. doi: 10.3389/fpls.2016.00310 (PMC4815178; doi:10.3389/fpls.2016.00310)
Supplement: Supplementary file 2 [file Table2.DOC]

**Supplementary Materials**

Additional Supplementary information may be found in the online version of this article

**Table S2 Analysis of AtHD2 protein components.**

| Protein type | Amino acids composition (%) | | | | | Secondary structure percentage (%) | | | | pI | MW  (KD) | Conserved domain | C- and N- end Hydrophobicity  and hydrophilism |
| --- | --- | --- | --- | --- | --- | --- | --- | --- | --- | --- | --- | --- | --- |
|  | Ile | Met | Phe | Thr | Tyr | Alpha-helix | Extended strand | β-turn | Random coil |  |  |  |  |
| AtHD2A | 2.0 | 0.8 | 2.9 | 4.9 | 1.2 | 21.22 | 19.18 | 9.8 | 49.8 | 5.12 | 26.37 | Zinc finger C2H2 | C- hydrophobicity  N-hydrophilism |
| AtHD2B | 2.0 | 0.7 | 2.9 | 5.6 | 0.3 | 9.8 | 18.63 | 6.21 | 65.36 | 4.68 | 32.35 | Low complexity region | C- hydrophobicity  N-hydrophilism |
| AtHD2C | 1.7 | 0.7 | 3.4 | 5.8 | 0.7 | 18.71 | 17.69 | 6.12 | 57.48 | 4.77 | 31.83 | Zinc finger,  Zinc finger C2H2 | C- hydrophobicity  N-hydrophilism |
| AtHD2D | 5.9 | 3.0 | 5.9 | 2.0 | 2.5 | 21.18 | 23.65 | 10.34 | 44.83 | 4.34 | 22.65 | Low complexity region | C- hydrophobicity  N-mainly with hydrophilism and part hydrophobicity |
