# Supplementary material for: AtHD2D Gene Plays a Role in Plant Growth, Development, and Response to Abiotic Stresses in Arabidopsis thaliana
Source: Front Plant Sci. 2016 Mar 31;7:310. doi: 10.3389/fpls.2016.00310 (PMC4815178; doi:10.3389/fpls.2016.00310)
Supplement: Supplementary file 3 [file Table3.DOC]

**Supplementary Materials**

Additional Supplementary information may be found in the online version of this article

**Table S3** **Prediction of the probable subcellular localization of signal peptide and AtHD2 proteins.**

| Contents | AtHD2A | AtHD2B | AtHD2C | AtHD2D |
| --- | --- | --- | --- | --- |
| Nucleus (%) | 60.9 | 65.2 | 73.9 | 17.4 |
| Cytoplasm (%) | 21.7 | 8.7 | 13.0 | 60.9 |
| Cytoskeleton (%) | 13.0 | 8.7 | 8.7 | 4.3 |
| Mitochondria (%) | 4.3 | 4.3 | 4.3 | 13.0 |
| Peroxysome (%) | - | - | - | 4.3 |
| Signal peptide | no | no | no | no |
| Integral prediction nuclear with score | 9.9 | 9.8 | 9.9 | 8.5 |
